# Multimodal physiological signal learning for clinical sleep respiratory event recognition

**DOI:** 10.3389/fneur.2026.1929036

**Published:** 2026-09-08

**Authors:** He Qin, Xiaocun Chen, Yao Liu, Xu Wei, Fei Chen, Lijie Lu, Liyun Liu, Tanjun Wei, Xionghui Hu, Xianhai Li, Xinhui Cheng

**Affiliations:** 1 Dazhou Integrated TCM & Western Medicine Hospital, Dazhou, China; 2 Chengdu Medical College, Chengdu, China

**Keywords:** cross-state token routing, event prototype fusion, multimodal physiological signals, sleep-related breathing event recognition, state-space model

## Abstract

Automatic recognition of sleep-related breathing events is of great significance for auxiliary sleep disorder screening, clinical interpretation, and long-term physiological monitoring. To address the limitations of existing methods in complementary modeling of multimodal physiological signals, event-related feature aggregation, and imbalanced class learning, this study proposes a multimodal state-space representation learning framework for sleep-related breathing event recognition. The framework takes the neurophysiological signal feature matrix and the respiration-acoustic-related feature matrix as inputs and employs a dual-branch state-space encoder to separately extract sequential dynamic features from different modalities. Furthermore, a cross-state token routing module is designed to realize fine-grained inter-modal information exchange, while an event query prototype fusion module aggregates event-related discriminative features from multimodal sequential representations. During training, class-balanced focal margin loss, sleep-stage auxiliary supervision, and arousal-state auxiliary supervision are jointly employed to improve the model's adaptability to complex sleep segments and imbalanced event distributions. Under patient-level five-fold cross-validation, the proposed method achieves an Accuracy of 88.19%, a Precision of 84.51%, a Recall of 86.28%, and an AUROC of 92.71% on the PSG-Audio public dataset. On the clinical dataset, it achieves an Accuracy of 74.33%, a Precision of 77.51%, a Recall of 84.33%, and an AUROC of 90.51%. These results indicate favorable overall performance of the proposed framework and provide supporting evidence for its ability to learn complementary multimodal physiological representations for sleep-related breathing event recognition.

## 1 Introduction

Sleep-related breathing disorders represent an important issue in public health and clinical sleep medicine. Their typical manifestations include recurrent apnea, hypopnea, oxygen saturation fluctuations, and arousal responses during sleep (1, 2). These events may not only disrupt sleep continuity and sleep architecture, but also be associated with daytime sleepiness, cognitive decline, increased cardiovascular and metabolic risks, and reduced quality of life. Therefore, accurate recognition of sleep-related breathing events is of great significance for disease screening, risk assessment, and clinical intervention (3). In current clinical practice, polysomnography remains an important basis for the evaluation of sleep-related breathing disorders. However, overnight recordings usually contain long-duration, multi-channel, and highly complex physiological signals. Manual scoring is time-consuming and can be affected by differences in clinical experience and uncertainty in event boundaries (4).

In recent years, deep learning methods have been gradually introduced into sleep physiological signal analysis tasks, showing certain potential in sleep staging, respiratory event detection, and abnormal state recognition (5, 6). Nevertheless, existing methods still have several limitations. First, some methods mainly rely on a single physiological modality, making it difficult to fully utilize the complementary information between neurophysiological state changes and respiration-related dynamics. Second, simple feature concatenation or late fusion strategies usually fail to characterize fine-grained interactions between different modalities at the levels of temporal segments and state transitions (7, 8). Third, sleep-related breathing events in real-world data often suffer from imbalanced class distributions, ambiguous event boundaries, and significant inter-individual differences, which may weaken the model's stable discriminative ability for complex samples (9). Therefore, how to construct a model framework that can simultaneously capture intra-modal dynamic dependencies, inter-modal interactions, and event-related discriminative structures remains a key problem in automatic sleep-related breathing event recognition.

To address the above issues, this study proposes a multimodal state-space representation learning framework for sleep-related breathing event recognition. The framework takes the neurophysiological signal feature matrix and the respiration-acoustic-related feature matrix as inputs, and employs a dual-branch state-space encoder to separately model long-range dynamic dependencies in different modalities. Based on this, a task-specific cross-state token routing module is developed by adapting established gated cross-attention mechanisms (10) to enable symmetric bidirectional information exchange between neurophysiological state representations and respiration-acoustic representations at the token level, thereby enhancing the complementary modeling capability between different modalities. Meanwhile, an event query prototype fusion module is developed by adapting learnable-query-based aggregation mechanisms (11) to respiratory-event-oriented multimodal temporal representations, allowing the model to actively aggregate discriminative information related to sleep-related breathing events. In addition, sleep stage and arousal state auxiliary supervision are incorporated to guide the model toward learning representations with stronger clinical context awareness. This design aims to improve the representation quality and event recognition stability of complex sleep segments, providing a more reasonable modeling strategy for automatic sleep-related breathing event analysis.

The main contributions of this study are summarized as follows:

- A multimodal state-space representation learning framework is proposed to jointly model neurophysiological state representations and respiration-acoustic-related representations, providing a unified sequential modeling scheme for sleep-related breathing event recognition.
- Task-specific CSTR and EQPF modules are developed by adapting established gated cross-attention and learnable-query mechanisms to sleep-related breathing event recognition. CSTR organizes symmetric bidirectional routing between neurophysiological and respiration-acoustic representations, whereas EQPF performs respiratory-event-oriented aggregation over multimodal temporal representations.
- A training objective combining class-balanced focal margin constraints and auxiliary clinical context supervision is constructed, enabling the model to better adapt to practical challenges such as class imbalance, ambiguous boundaries, and inter-individual differences.

## 2 Related work

### 2.1 Automated sleep respiratory event detection in clinical physiological monitoring

Automatic detection of sleep-related breathing events is an important research direction in clinical sleep medicine and physiological monitoring. Its core objective is to automatically identify normal breathing, hypopnea, obstructive sleep apnea, and other abnormal respiratory events in polysomnography, home sleep testing, or portable physiological monitoring scenarios, thereby reducing the burden of manual scoring and improving diagnostic consistency. Nasiri et al. proposed the CAISR system, which achieved automated sleep analysis covering multiple clinical sleep metrics and demonstrated the application potential of artificial intelligence methods in clinical-grade sleep event scoring (12). Massie et al. proposed a context-aware automatic scoring method for home sleep apnea detection, emphasizing the use of event contextual information to improve automatic scoring accuracy (13). Yook et al. proposed a deep learning-based method for learning sleep apnea–hypopnea events, which was used for obstructive sleep apnea classification and clinical severity assessment (14). Retamales et al. proposed an automatic sleep apnea estimation method for home-based scenarios, further promoting the application of deep learning models in non-laboratory sleep monitoring (15). Alarcón et al. developed an interpretable deep learning model for obstructive sleep apnea event detection using portable monitoring devices, focusing on model interpretability and clinical usability (16). Dang et al. proposed an automatic obstructive sleep apnea detection algorithm based on a wireless abdominal wearable sensor, demonstrating the practical application value of wearable physiological monitoring devices in sleep-related breathing event recognition (17).

In recent years, automatic recognition of sleep-related breathing events has gradually shifted from single-signal analysis toward multi-task learning, multimodal physiological representation fusion, and interpretable clinical decision support. Wang et al. proposed a multi-task learning method that integrates obstructive sleep apnea detection and sleep staging, using multi-scale modeling to simultaneously capture respiratory event patterns and sleep-stage-related information (18). García-Vicente et al. proposed SleepECG-Net, an interpretable deep learning approach for pediatric sleep apnea diagnosis using electrocardiographic signals, highlighting the value of single-channel physiological signals for screening special populations (19). Barroso-García et al. proposed an explainable detection method for pediatric sleep apnea based on single-channel airflow signals, further validating the feasibility of lightweight physiological monitoring signals for automatic diagnosis (20). D'Orazio et al. proposed the REST platform for simultaneous interpretable detection of apnea, oxygen desaturation, and artifacts in pediatric polysomnographic monitoring, indicating that automated systems can support both event recognition and signal quality control (21). Hemrajani et al. proposed SleepNet, which integrates multiple physiological signals to improve sleep apnea diagnostic performance, reflecting the advantages of multimodal physiological information fusion in the assessment of complex sleep disorders (22). Ban et al. proposed a breathable flexible bioelectronic device to enhance the automatic detection of obstructive sleep apnea, suggesting that the combination of emerging physiological sensing technologies and intelligent algorithms is expanding the application boundaries of clinical sleep monitoring (23).

### 2.2 Multimodal physiological representation learning for sleep disorder assessment

Multimodal physiological representation learning has become an important research direction in sleep disorder assessment. Its core idea is to jointly utilize multi-source information, including electroencephalography, electrooculography, electromyography, electrocardiography, respiratory signals, blood oxygen saturation, and wearable sensor signals, to characterize sleep-state changes and abnormal event patterns from different physiological dimensions. Lee et al. proposed an explainable Vision Transformer model for automatic sleep staging using multimodal polysomnographic signals, and enhanced the interpretability of the model in clinical sleep analysis through an explainable mechanism (24). Xu et al. proposed a sleep staging method that integrates multimodal features with a denoising diffusion model, improving the representational complementarity among different physiological signals through generative modeling (25). Duan et al. proposed MMS-SleepNet, which combines knowledge-guided mechanisms, multimodal fusion, and multi-scale network structures to capture complex physiological changes related to sleep stages (26). Wang et al. proposed MASleepNet, which integrates multi-scale convolution and attention mechanisms into a sleep staging model to enhance the joint modeling capability of local rhythmic patterns and global temporal dependencies (27). Zheng et al. proposed MMASleepNet, a multimodal attention network based on electrophysiological signals for automatic sleep staging, demonstrating that attention mechanisms can effectively improve the discriminative aggregation ability of multi-source physiological signals (28).

In addition to traditional polysomnographic signals, recent studies have also begun to focus on sleep disorder assessment under wearable, multi-sensor, and temporal fusion scenarios. Kucukosmanoglu et al. proposed a method for detecting cortical arousal events during sleep using multimodal wearable sensors and machine learning, indicating that lightweight physiological monitoring signals can provide effective evidence for sleep interruption and sleep quality assessment (29). Pan et al. proposed MMTSleepNet, which realizes multimodal fusion in a time-series manner to enhance dynamic modeling capability during sleep-stage transitions (30). Gui et al. proposed MFSleepNet, an interactive multimodal fusion framework designed to improve information interaction among different physiological modalities and enhance sleep staging performance (31). Ali et al. proposed a sleep stage classification method based on multi-sensor wearable data and federated learning, enabling the modeling of multi-source sleep physiological information while preserving privacy (32). Duan et al. earlier proposed a sleep staging network that combines data adaptation and multimodal feature fusion, laying a foundation for the subsequent development of multimodal physiological representation learning in sleep disorder assessment (33).

## 3 Method

### 3.1 Problem definition

This study focuses on the task of binary sleep-related breathing event recognition in clinical sleep monitoring scenarios. Given a dataset containing *N* sleep segments, denoted as $D={\left\{\left({\text{X}}_{i}^{n},{\text{X}}_{i}^{r},y_{i},s_{i},a_{i}\right)\right\}}_{i=1}^{N}$, where ${\text{X}}_{i}^{n}\in {\mathbb{R}}^{T\times C_{n}}$ represents the neurophysiological signal feature matrix of the *i*-th sample, which is used to characterize the dynamic representations of electroencephalographic, electrooculographic, or electromyographic activities related to sleep states; ${\text{X}}_{i}^{r}\in {\mathbb{R}}^{T\times C_{r}}$ represents the physiological signal feature matrix related to respiratory and acoustic activities, which is used to describe respiratory rhythm, airflow variation, and abnormal breathing patterns. Here, *T* denotes the temporal length, while *C*_*n*_ and *C*_*r*_ denote the feature dimensions of different modalities, respectively. The primary task label is denoted as *y*_*i*_∈{0, 1}, where *y*_*i*_ = 0 indicates a normal sleep segment and *y*_*i*_ = 1 indicates an abnormal sleep-related breathing event segment. In addition, *s*_*i*_∈{0, 1, …, *S*−1} denotes the sleep stage label, and *a*_*i*_∈{0, 1} indicates whether an arousal response is present. The objective of this study is to learn a multimodal discriminative function $f_{\theta }:\left({\text{X}}_{i}^{n},{\text{X}}_{i}^{r}\right)\to {ŷ}_{i}$. By jointly modeling the complementary relationship between neurophysiological state representations and respiration-related representations, the proposed framework aims to achieve accurate binary recognition of sleep-related breathing events. Meanwhile, sleep stages and arousal responses are used as auxiliary supervision signals to guide the model toward learning multimodal sequential representations with stronger clinical context awareness.

### 3.2 Overall model architecture

**Figure 1:**
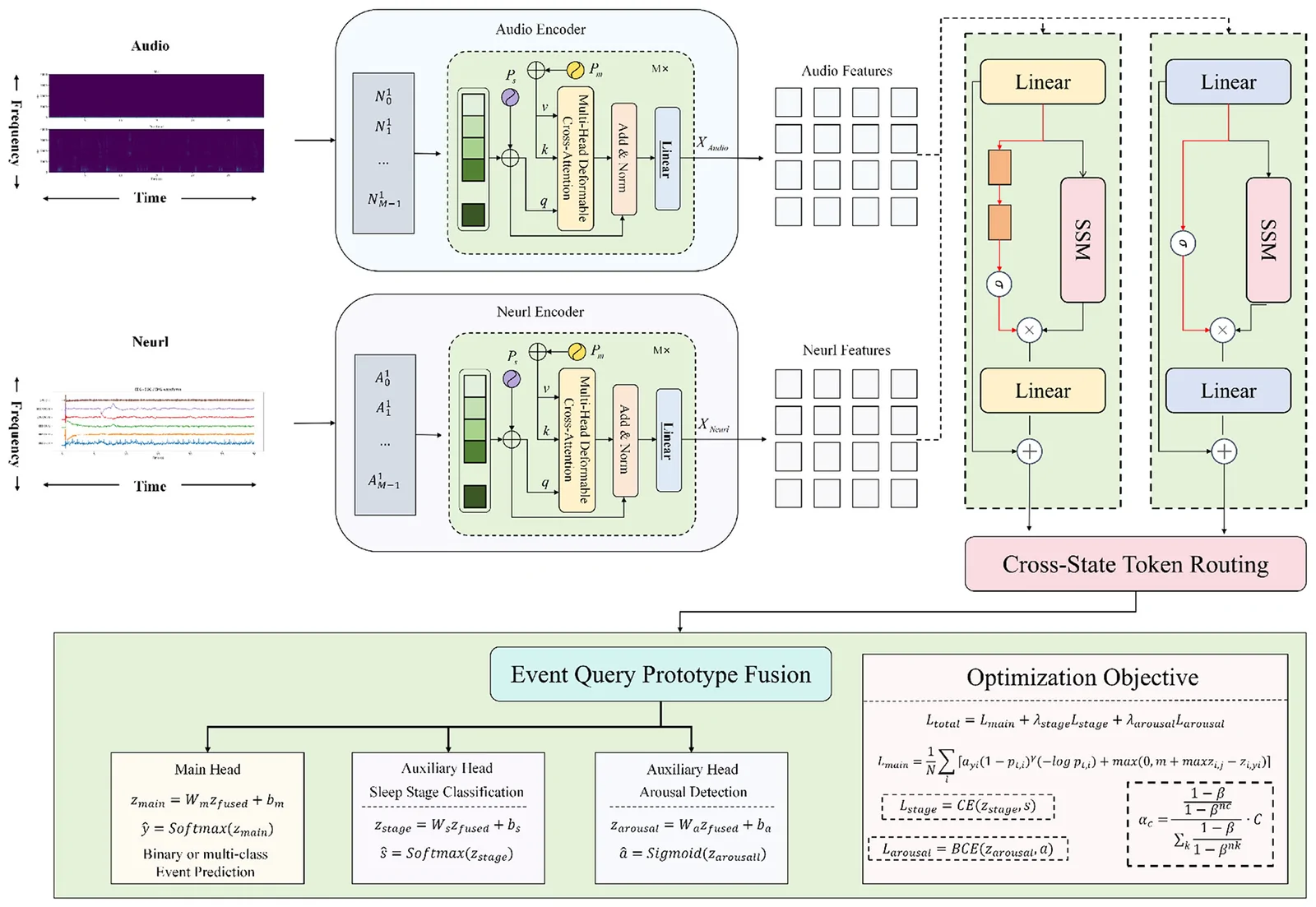
The proposed multimodal sleep-related breathing event recognition framework consists of a neurophysiological encoder, a respiration-acoustic encoder, a cross-state token routing module, and an event query prototype fusion module. It is designed to jointly model long-range dynamic dependencies and event-related discriminative information across different physiological modalities. In addition to the primary respiratory event classification task, the model introduces auxiliary branches for sleep stage classification and arousal detection, thereby enhancing the clinical context awareness of multimodal sequential representations.

The overall framework proposed in this study realizes sleep-related breathing event recognition through three hierarchical components: dual-branch state-space encoding, multimodal cross-state interaction, and event prototype aggregation. This design preserves both the sleep-stage context embedded in neurophysiological state representations and the abnormal rhythmic patterns contained in respiration-related representations. Compared with directly concatenating features from different modalities, the proposed framework models inter-modal dependencies at the sequence-token level, thereby obtaining multimodal representations with stronger event-discriminative capability. The overall model architecture is shown in Figure 1.

Specifically, given the neurophysiological signal feature matrix ${\text{X}}_{i}^{n}$ and the respiration-acoustic-related feature matrix ${\text{X}}_{i}^{r}$ of the *i*-th sample, the model first employs two modality-specific state-space encoders to extract intra-modal long-range dependencies. Here, *m*∈{*n, r*} denotes different modalities, $T_{m}\left(\cdot \right)$ denotes the sequence embedding mapping, **P**_*m*_ denotes the learnable positional representation, and $E_{m}\left(\cdot \right)$ denotes the corresponding state-space encoder:


$$\begin{array}{lll} {\text{Z}}_{i}^{m} & = & E_{m}\left(T_{m}\left({\text{X}}_{i}^{m}\right)+{\text{P}}_{m}\right)=\left[{\text{z}}_{i,0}^{m},{\text{z}}_{i,1}^{m},\ldots ,{\text{z}}_{i,L}^{m}\right]\in {\mathbb{R}}^{\left(L+1\right)\times D},\\ m & \in & \left\{n,r\right\}. \end{array}$$ (1)

Here, ${\text{Z}}_{i}^{n}$ and ${\text{Z}}_{i}^{r}$ represent the neurophysiological token sequence and the respiration-acoustic token sequence, respectively. ${\text{z}}_{i,0}^{m}$ denotes the global state token, *L* is the number of sequence tokens, and *D* is the hidden feature dimension. Subsequently, a cross-state token routing module $R_{\phi }\left(\cdot \right)$ is introduced to establish bidirectional information transmission between neurophysiological state tokens and respiration-acoustic state tokens, enabling the two modalities to achieve adaptive alignment and complementary enhancement over event-related temporal segments:


$$\begin{array}{l} \left({\tilde{\text{Z}}}_{i}^{n},{\tilde{\text{Z}}}_{i}^{r}\right)=R_{\phi_{2}}\left(R_{\phi_{1}}\left({\text{Z}}_{i}^{n},{\text{Z}}_{i}^{r}\right)\right). \end{array}$$ (2)

Here, $R_{\phi_{1}}\left(\cdot \right)$ and $R_{\phi_{2}}\left(\cdot \right)$ denote the first and second Cross-State Token Routing blocks, respectively, and ϕ_1_ and ϕ_2_ represent their learnable parameters. The outputs ${\tilde{\text{Z}}}_{i}^{n}$ and ${\tilde{\text{Z}}}_{i}^{r}$ are the neurophysiological and respiration-acoustic token sequences enhanced through the two successive routing blocks. After obtaining the cross-modality-enhanced token sequences, this study further designs an event query prototype fusion module to actively aggregate discriminative information related to sleep-related breathing events from multimodal sequential representations. Let ${\text{Q}}_{e}\in {\mathbb{R}}^{K\times D}$ denote the learnable event query prototypes, where *K* is the number of respiratory event categories. Attn(·) denotes the query-based cross-token attention reading operation, Pool(·) denotes the event prototype aggregation operation, and $F_{\psi }\left(\cdot \right)$ denotes the fusion mapping function. The final multimodal event representation **h**_*i*_ is formulated as:


$$\begin{array}{l} {\text{h}}_{i}=F_{\psi }\left(\left[{\tilde{\text{z}}}_{i,0}^{n};{\tilde{\text{z}}}_{i,0}^{r};\mathrm{Pool}\left(\mathrm{Attn}\left({\text{Q}}_{e},\left[{\tilde{\text{Z}}}_{i}^{n};{\tilde{\text{Z}}}_{i}^{r}\right]\right)\right)\right]\right). \end{array}$$ (3)

Finally, **h**_*i*_ is simultaneously fed into the main classification head, the sleep-stage auxiliary head, and the arousal detection auxiliary head, producing the sleep-related breathing event prediction ŷ_*i*_, the sleep-stage prediction ŝ_*i*_, and the arousal response prediction â_*i*_, respectively. The main classification head distinguishes normal sleep segments from abnormal sleep-related breathing event segments. The two auxiliary heads provide more stable clinical contextual constraints for the main task through supervision from sleep stages and arousal states.

### 3.3 Cross-state token routing

**Figure 2:**
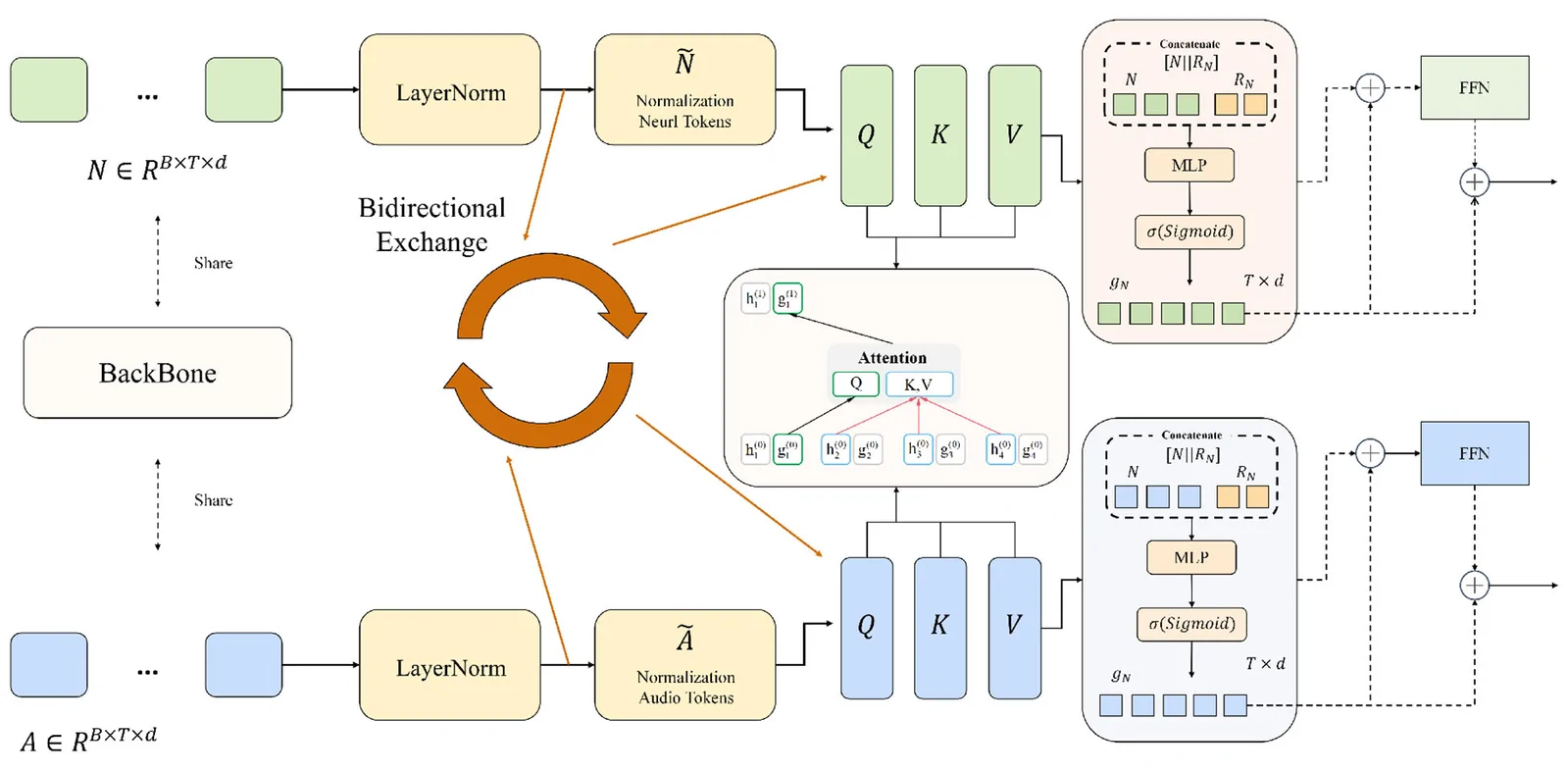
The Cross-State Token Routing module achieves fine-grained state exchange and adaptive information fusion between neurophysiological tokens and respiration-acoustic tokens through bidirectional cross-modal attention and gated residual updating.

To further enhance the interaction capability between neurophysiological state representations and respiration-acoustic representations, this study develops a task-specific cross-state token routing module by adapting established gated cross-attention mechanisms in multimodal representation learning (10). The contribution of CSTR does not lie in introducing the basic cross-attention or gating operations themselves, but in organizing them as symmetric bidirectional routing paths between neurophysiological and respiration-acoustic token sequences, together with modality-specific gated residual integration for sleep-related breathing event recognition. Compared with multimodal sleep-staging models such as MMASleepNet (28), CSTR is not designed as a generic feature-fusion operation for sleep-stage prediction. Instead, it performs token-level information routing in both directions, uses modality-specific token-wise and channel-wise gates to regulate the amount of heterogeneous information injected into each branch, and preserves the original modality representations through independent residual pathways. Therefore, its task-specific distinction lies in explicitly modeling the complementary relationship between neurophysiological states and respiration-acoustic abnormalities rather than simply applying cross-modal attention to globally fused features. Instead of simply concatenating the two modalities, this module establishes bidirectional information transmission paths in the normalized sequence-token space. In this way, respiration-related abnormal patterns can be fed back into neurophysiological state modeling, while sleep-stage-related contextual information can inversely regulate respiratory event representations. The architecture of this module is shown in Figure 2.

Let the neurophysiological token sequence and the respiration-acoustic token sequence obtained from the previous stage be denoted as ${\text{Z}}_{i}^{n}\in {\mathbb{R}}^{\left(L+1\right)\times D}$ and ${\text{Z}}_{i}^{r}\in {\mathbb{R}}^{\left(L+1\right)\times D}$, respectively, where *L* denotes the number of local sequence tokens and *D* denotes the hidden feature dimension. The two modalities are first normalized within their respective modal spaces:


$$\begin{array}{l} {\bar{\text{Z}}}_{i}^{m}={\mathrm{LN}}_{m}\left({\text{Z}}_{i}^{m}\right),\;m\in \left\{n,r\right\}. \end{array}$$ (4)

In the normalized token space, query, key, and value mappings are constructed for both the neurophysiological modality and the respiration-acoustic modality. Different from a single or asymmetric cross-modal attention operation, cross-state token routing adopts a symmetric bidirectional exchange mechanism, allowing each modality to actively read event-related information from the other modality as the query side. Specifically, ${\text{W}}_{Q}^{m}$, ${\text{W}}_{K}^{m}$, and ${\text{W}}_{V}^{m}$ denote the linear projection matrices corresponding to modality *m*. The query, key, and value tokens are formulated as:


$$\begin{array}{l} {\text{Q}}_{i}^{m}={\bar{\text{Z}}}_{i}^{m}{\text{W}}_{Q}^{m},\;{\text{K}}_{i}^{m}={\bar{\text{Z}}}_{i}^{m}{\text{W}}_{K}^{m},\;{\text{V}}_{i}^{m}={\bar{\text{Z}}}_{i}^{m}{\text{W}}_{V}^{m},\;m\in \left\{n,r\right\}. \end{array}$$ (5)

Subsequently, the module realizes state-token exchange through bidirectional cross-modal attention. For the neurophysiological branch, the neurophysiological query ${\text{Q}}_{i}^{n}$ is used as the retrieval entry to read respiration-event-related information from the respiration-acoustic key-value pair $\left({\text{K}}_{i}^{r},{\text{V}}_{i}^{r}\right)$, thereby obtaining the respiratory routing representation injected into the neurophysiological state space:


$$\begin{array}{l} {\text{R}}_{i}^{n}=\mathrm{Softmax}\left(\frac{{\text{Q}}_{i}^{n}{\left({\text{K}}_{i}^{r}\right)}^{⊤}}{\sqrt{D}}\right){\text{V}}_{i}^{r}. \end{array}$$ (6)

Correspondingly, for the respiration-acoustic branch, the respiration-acoustic query ${\text{Q}}_{i}^{r}$ is used as the retrieval entry to read sleep-state-related context from the neurophysiological key-value pair $\left({\text{K}}_{i}^{n},{\text{V}}_{i}^{n}\right)$, thereby obtaining the neurophysiological state routing representation injected into the respiratory representation space:


$$\begin{array}{l} {\text{R}}_{i}^{r}=\mathrm{Softmax}\left(\frac{{\text{Q}}_{i}^{r}{\left({\text{K}}_{i}^{n}\right)}^{⊤}}{\sqrt{D}}\right){\text{V}}_{i}^{n}. \end{array}$$ (7)

To avoid excessive injection of cross-modal information, this study further introduces a gated modulation mechanism to adaptively filter the routed heterogeneous-modal information. Specifically, the module concatenates the original modality tokens with the corresponding cross-modal routing tokens along the feature dimension and applies the same multilayer perceptron independently to each token position. This generates token-wise and channel-wise gating weights, enabling the model to dynamically determine the fusion intensity of cross-modal information according to the physiological state of different sleep segments:


$$\begin{array}{l} {\text{G}}_{i,t}^{m}=\sigma \left({\mathrm{MLP}}_{m}\left(\left[{\text{Z}}_{i,t}^{m};{\text{R}}_{i,t}^{m}\right]\right)\right),\;t=0,\ldots ,L,\;m\in \left\{n,r\right\}, \end{array}$$ (8)

where ${\text{Z}}_{i,t}^{m},{\text{R}}_{i,t}^{m}\in {\mathbb{R}}^{D}$ denote the original and routed representations at token position *t*, respectively. Consequently, ${\text{G}}_{i}^{m}\in {\mathbb{R}}^{\left(L+1\right)\times D}$ is obtained by stacking the gating vectors from all token positions. Finally, the gated cross-state routing information is injected into the original modality sequence through residual connection and further processed by a feed-forward network for intra-modal feature reorganization. The enhanced neurophysiological token sequence ${\tilde{\text{Z}}}_{i}^{n}$ and respiration-acoustic token sequence ${\tilde{\text{Z}}}_{i}^{r}$ are obtained as:


$$\begin{array}{l} {\tilde{\text{Z}}}_{i}^{m}={\text{Z}}_{i}^{m}+{\text{G}}_{i}^{m}⊙{\text{R}}_{i}^{m}+{\mathrm{FFN}}_{m}\left({\text{Z}}_{i}^{m}+{\text{G}}_{i}^{m}⊙{\text{R}}_{i}^{m}\right),\;m\in \left\{n,r\right\}. \end{array}$$ (9)

Through the above design, the cross-state token routing module establishes fine-grained bidirectional complementary relationships while preserving modality-specific representations, thereby providing more discriminative multimodal sequential representations for subsequent event query prototype fusion.

### 3.4 Event query prototype fusion

**Figure 3:**
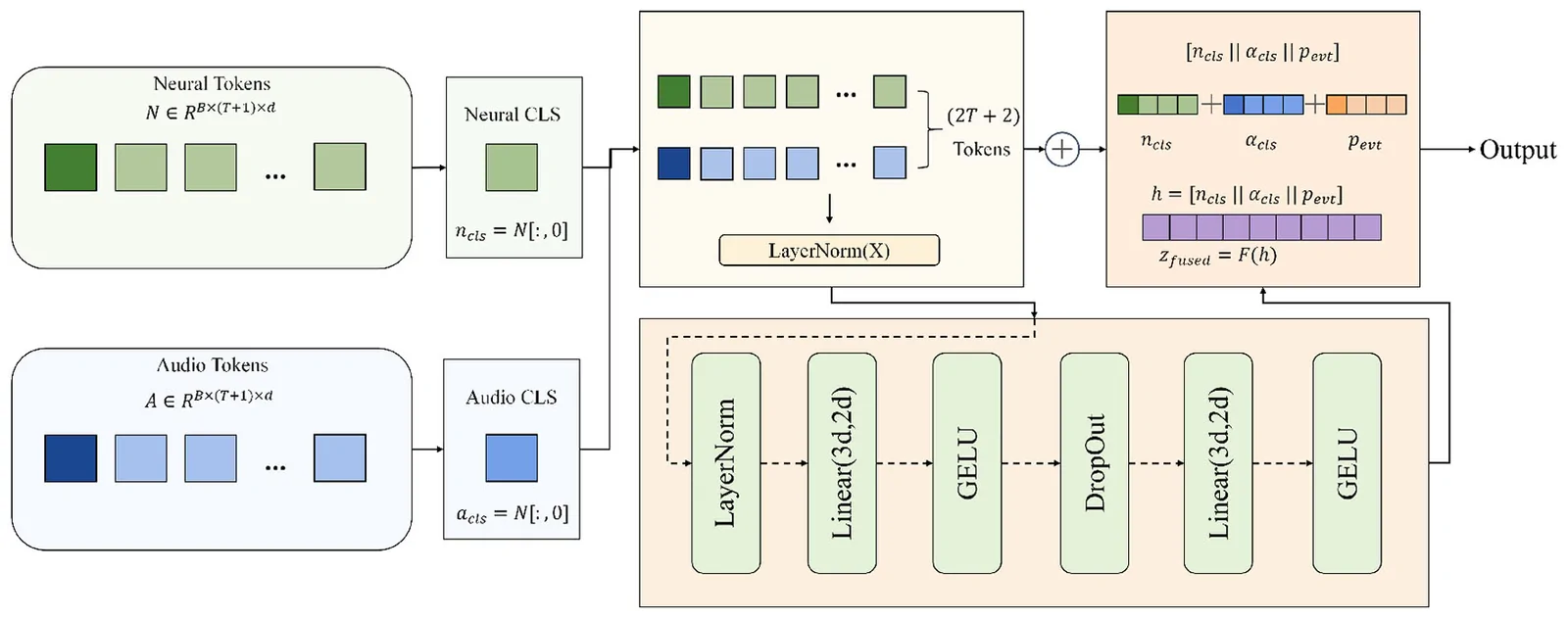
The Event Query Prototype Fusion module extracts global state tokens from neurophysiological and respiration-acoustic modalities, and combines event query prototypes to discriminatively aggregate multimodal sequence tokens, thereby generating fused representations for sleep-related breathing event recognition.

After obtaining the enhanced neurophysiological token sequence ${\tilde{\text{Z}}}_{i}^{n}$ and respiration-acoustic token sequence ${\tilde{\text{Z}}}_{i}^{r}$ from the cross-state token routing module, this study further designs an event query prototype fusion module to actively aggregate discriminative information related to sleep-related breathing events from multimodal sequential representations. The use of learnable queries in EQPF is conceptually related to the object-query mechanism introduced in DETR (11). However, EQPF does not perform object-set prediction or spatial localization. Instead, it interprets the learnable queries as respiratory-event prototypes that retrieve category-relevant information from the combined neurophysiological and respiration-acoustic temporal token pool. Different from directly using global average pooling or simple concatenation, this module explicitly preserves the global state tokens of the two modalities and introduces learnable event query prototypes to perform event-aware reading over all sequence tokens. The architecture of this module is shown in Figure 3.

First, the corresponding global state tokens are extracted from the enhanced sequences of the two modalities:


$$\begin{array}{l} {\text{c}}_{i}^{n}={\tilde{\text{Z}}}_{i}^{n}\left[0\right],\;{\text{c}}_{i}^{r}={\tilde{\text{Z}}}_{i}^{r}\left[0\right]. \end{array}$$ (10)

Here, ${\text{c}}_{i}^{n}$ denotes the neurophysiological global state representation, and ${\text{c}}_{i}^{r}$ denotes the respiration-acoustic global state representation. Subsequently, the complete token sequences of the two modalities are concatenated, and a unified multimodal event candidate token pool is constructed through normalization:


$$\begin{array}{l} {\text{U}}_{i}={\mathrm{LN}}_{e}\left(\left[{\tilde{\text{Z}}}_{i}^{n};{\tilde{\text{Z}}}_{i}^{r}\right]\right)\in {\mathbb{R}}^{2\left(L+1\right)\times D}. \end{array}$$ (11)

To enable the model to form more explicit discriminative centers around different respiratory event types, this study defines learnable event query prototypes ${\text{Q}}_{e}\in {\mathbb{R}}^{K\times D}$, where *K* denotes the number of respiratory event categories and *D* denotes the hidden feature dimension. For the *i*-th sample, the event query prototypes are expanded into a sample-level query matrix and normalized as the active retrieval entry, which is used to read event-related representations from the multimodal token pool:


$$\begin{array}{l} {\text{E}}_{i}={\mathrm{LN}}_{q}\left({\text{Q}}_{e}\right)\in {\mathbb{R}}^{K\times D}. \end{array}$$ (12)

Based on this event query matrix, the module calculates the attention associations between event prototypes and multimodal tokens, thereby obtaining the response intensity of different event prototypes to neurophysiological states and respiration-acoustic states:


$$\begin{array}{l} {\text{A}}_{i}^{e}=\mathrm{Softmax}\left(\frac{{\text{E}}_{i}{\text{U}}_{i}^{⊤}}{\sqrt{D}}\right)\in {\mathbb{R}}^{K\times 2\left(L+1\right)}. \end{array}$$ (13)

After obtaining the event attention weights, this study uses them to perform weighted reading over the multimodal token pool, producing class-prototype-level event response representations. This process can be regarded as using *K* learnable event queries to simultaneously retrieve abnormal respiratory patterns from neurophysiological dynamics and respiration-acoustic dynamics. As a result, the model not only focuses on a single global state but also aggregates fine-grained information related to event types from local sequence tokens:


$$\begin{array}{l} {\text{P}}_{i}^{e}={\text{A}}_{i}^{e}{\text{U}}_{i}\in {\mathbb{R}}^{K\times D}. \end{array}$$ (14)

Furthermore, to form a compact sample-level event prototype representation, the module aggregates the reading results of all event queries and obtains the final event-aware prototype vector:


$$\begin{array}{l} {\text{p}}_{i}^{e}=\mathrm{Pool}\left({\text{P}}_{i}^{e}\right)=\frac{1}{K}\overset{K}{\underset{k=1}{\sum }}{\text{P}}_{i,k}^{e}\in {\mathbb{R}}^{D}. \end{array}$$ (15)

Finally, this study fuses the neurophysiological global state token, the respiration-acoustic global state token, and the event-aware prototype vector to construct a comprehensive representation that simultaneously contains sleep-state context, respiratory rhythm variations, and event-discriminative centers. Let $F_{\psi }\left(\cdot \right)$ denote the fusion mapping function composed of normalization, linear mapping, nonlinear activation, and dropout. The final multimodal event representation can be written as:


$$\begin{array}{l} {\text{h}}_{i}=\left[{\text{c}}_{i}^{n};{\text{c}}_{i}^{r};{\text{p}}_{i}^{e}\right]\in {\mathbb{R}}^{3D}. \end{array}$$ (16)

$$\begin{array}{l} {\text{z}}_{i}^{fused}=F_{\psi }\left({\text{h}}_{i}\right)\in {\mathbb{R}}^{D}. \end{array}$$ (17)

Here, ${\text{z}}_{i}^{fused}$ serves as the shared input representation for the subsequent main respiratory event classification head, sleep-stage auxiliary head, and arousal detection auxiliary head. Through the above design, the event query prototype fusion module further highlights event-related information based on cross-modality-enhanced representations, enabling the final features to contain not only modality complementarity but also a category-discriminative structure oriented toward respiratory event recognition. Accordingly, the task-specific contribution of EQPF lies in combining respiratory-event-oriented temporal retrieval with the global state representations of both physiological modalities, rather than in introducing the learnable-query operation itself.

### 3.5 Training objective and loss function

To enable the model to simultaneously possess class-discriminative ability, clinical context awareness, and robustness to imbalanced samples in the task of sleep-related breathing event recognition, this study adopts a multi-task joint optimization strategy. Given the fused multimodal event representation ${\text{z}}_{i}^{fused}$, the main classification head outputs the respiratory event prediction logits ${\text{o}}_{i}^{main}\in {\mathbb{R}}^{K}$, the sleep-stage auxiliary head outputs ${\text{o}}_{i}^{stage}\in {\mathbb{R}}^{S}$, and the arousal detection auxiliary head outputs $o_{i}^{arousal}\in \mathbb{R}$. Here, the main task label is denoted as *y*_*i*_, the sleep stage label as *s*_*i*_, and the arousal label as *a*_*i*_. Considering that normal segments and abnormal respiratory events usually exhibit obvious class imbalance in clinical data, this study first constructs class-balanced weights according to the number of samples *n*_*c*_ in each class of the training set:


$$\begin{array}{l} \alpha_{c}=\frac{\left(1-\beta \right)/\left(1-\beta^{n_{c}}\right)}{\overset{K-1}{\underset{j=0}{\sum }}\left(1-\beta \right)/\left(1-\beta^{n_{j}}\right)}\cdot K. \end{array}$$ (18)

For the main respiratory event recognition task, this study further combines a focal modulation term and a margin constraint term to construct a class-balanced focal margin loss. Here, *p*_*i*,_*y*__*i*__ denotes the predicted probability of sample *i* on its ground-truth class *y*_*i*_, γ is the focal modulation coefficient, δ is the margin coefficient, and $\underset{j\neq y_{i}}{\max}o_{i,j}^{main}$ denotes the logit corresponding to the hardest negative class:


$$\begin{array}{lll} L_{main} & = & \frac{1}{N}\overset{N}{\underset{i=1}{\sum }}[\alpha_{y_{i}}{(1-p_{i,y_{i}})}^{\gamma }(-\log p_{i,y_{i}})\\ + & \max(0,\delta +\underset{j\neq y_{i}}{\max}\overset{main}{\underset{i,j}{o}}-\overset{main}{\underset{i,y_{i}}{o}})]. \end{array}$$ (19)

In addition to the main classification objective, this study uses sleep stage recognition and arousal detection as auxiliary supervision signals to constrain the model to learn more stable sleep-state context and event-associated response representations. Specifically, sleep stage classification adopts the cross-entropy loss CE(·), while arousal detection adopts the binary cross-entropy loss BCE(·). The final training objective consists of the weighted combination of the main task loss, the sleep-stage auxiliary loss, and the arousal auxiliary loss:


$$\begin{array}{l} L_{total}=L_{main}+\lambda_{stage}\mathrm{CE}\left({\text{o}}_{i}^{stage},s_{i}\right)+\lambda_{arousal}\mathrm{BCE}\left(o_{i}^{arousal},a_{i}\right). \end{array}$$ (20)

The overall training process is shown in Algorithm 1. The model first reads the neurophysiological signal feature matrix and the respiration-acoustic-related feature matrix. After dual-branch state-space encoding, cross-state token routing, and event query prototype fusion, a shared multimodal representation is obtained. Then, the outputs of the main task and auxiliary tasks are computed separately, and all learnable parameters are updated through back-propagation with the joint loss.

Algorithm 1 Training procedure of the multimodal sleep-related breathing event recognition model. 
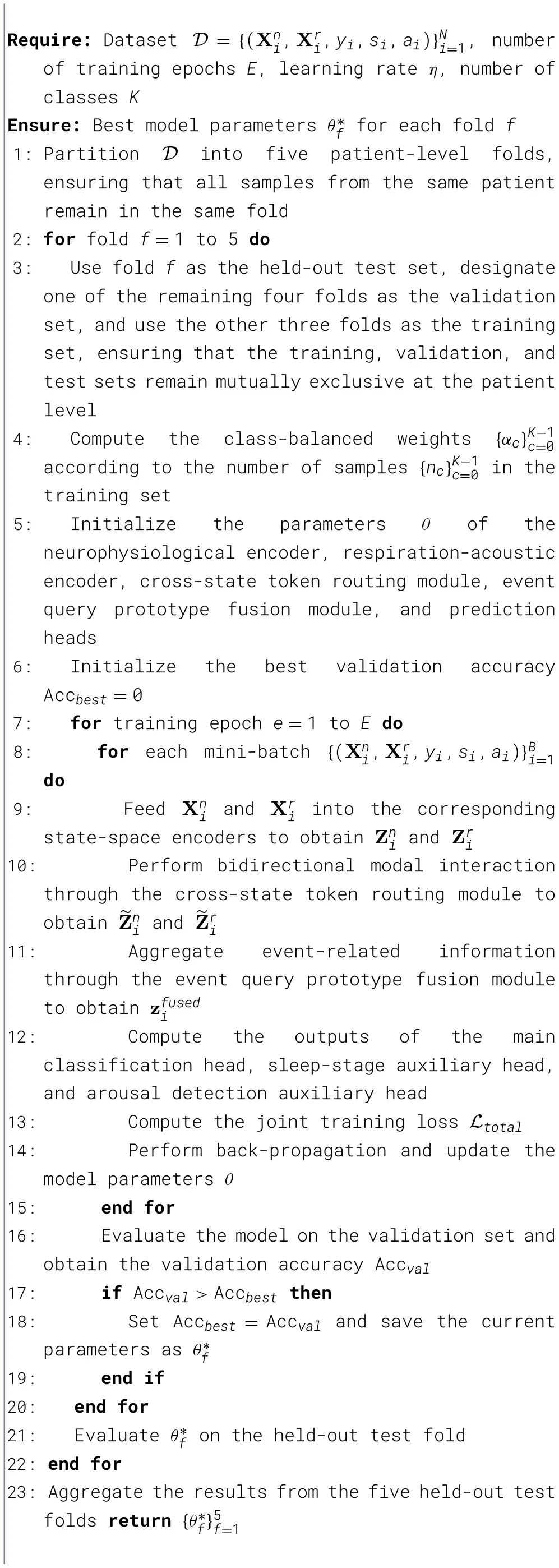

## 4 Datasets and evaluation metrics

### 4.1 Dataset

#### 4.1.1 Public datasets

This study uses the public PSG-Audio sleep-related breathing event dataset as the experimental data source. This dataset contains overnight polysomnographic recordings and synchronized respiratory acoustic signals from multiple subjects, and provides manually annotated sleep stages and respiratory event information. Based on the original EDF signal files and RML annotation files, each sleep recording is divided into fixed-length 30 s sleep segments. Neurophysiological modality features and respiration-acoustic modality features are then extracted separately. The neurophysiological modality mainly includes EEG-, EOG-, and EMG-related signals, while the respiration-acoustic modality mainly includes Mic- and Tracheal-related signals. For the binary respiratory event recognition task, segments without annotated respiratory events are labeled as Normal, while segments overlapping with Hypopnea, ObstructiveApnea, MixedApnea, or CentralApnea events are labeled as Abnormal. Each sleep segment is assigned a binary respiratory event label, a sleep stage label, and an arousal status label.

To avoid data leakage caused by samples from the same patient appearing in different data partitions, this study adopts patient-level five-fold cross-validation. All segments belonging to the same patient are assigned to the same fold. The subjects are partitioned into five mutually exclusive folds. In each cross-validation round, four folds are used as the development set for model training and validation, while the remaining fold is held out as the test set. This procedure is repeated five times so that each fold serves as the test set once, and the final performance is obtained by aggregating the results across the five test folds. The detailed experimental setup is shown in Table 1.

**Table 1:** Public dataset and preprocessing settings.

Item	Description
Dataset name	PSG-Audio public sleep-related breathing event dataset
Data source	Public data resource from Science Data Bank
Data scale	Overnight sleep monitoring records from 212 subjects
Original files	EDF physiological signal files and RML event annotation files
Segment length	30 s non-overlapping sleep segments
Neurophysiological modality	EEG-, EOG-, and EMG-related signals
Respiration-acoustic modality	Mic- and Tracheal-related signals
Main task labels	Normal and Abnormal respiratory event segments
Abnormal event definition	Segments overlapping with Hypopnea, ObstructiveApnea, MixedApnea, or CentralApnea events
Auxiliary labels	Sleep stage and arousal status
Data splitting strategy	Patient-level five-fold cross-validation, with all segments from the same patient retained in the same fold
Cross-validation protocol	Four folds are used for model training and validation, and the remaining fold is used for testing; each fold serves as the test set once

#### 4.1.2 Clinical dataset

This study further constructed a clinical sleep monitoring dataset using retrospectively collected records from Dazhou Integrated Traditional Chinese and Western Medicine Hospital. The dataset included 83 patients and was used to evaluate the proposed method in a clinical setting. The original recordings were divided into 30-s non-overlapping segments. Segments without definite sleep-related breathing abnormalities were labeled as normal, whereas segments containing respiratory obstruction-related events were labeled as abnormal.

As shown in Table 2 of 49.73 ± 12.68 years, a mean body mass index of 27.84 ± 4.31 kg/m^2^, and a mean apnea–hypopnea index of 31.62 ± 18.47 events/hour. A total of 54,370 segments were included, comprising 21,476 normal segments and 32,894 abnormal segments. The corresponding normal-to-abnormal ratio was approximately 1:1.53, indicating a moderate class imbalance.

**Table 2:** Clinical characteristics and class distribution of the clinical dataset.

Characteristic	Value
Number of patients	83
Age, years	49.73 ± 12.68
BMI, kg/m^2^	27.84 ± 4.31
AHI, events/hour	31.62 ± 18.47
Segment duration	30 s, non-overlapping
Total number of segments	54,370
Normal segments	21,476 (39.50%)
Abnormal segments	32,894 (60.50%)
Normal-to-abnormal ratio	1:1.53
Number of independent annotators	2
Inter-rater agreement	Cohen's κ = 0.79
Cross-validation strategy	Patient-level five-fold cross-validation

The clinical dataset was processed using the same preprocessing procedure as the public dataset. Patient-level five-fold cross-validation was adopted, and all segments belonging to the same patient were retained within the same fold. In each cross-validation round, one fold was used as the held-out test set, while the remaining folds were used for model development.

The segment annotations were independently performed by two experts with experience in sleep monitoring and respiratory event interpretation. The initial inter-rater agreement before consensus review was assessed using Cohen's κ, which was 0.79. Segments with inconsistent initial annotations were subsequently reviewed and discussed to determine the final labels. All clinical data were de-identified before analysis. The study was approved by the Medical Ethics Committee of Dazhou Integrated Traditional Chinese and Western Medicine Hospital under approval number 202509. The requirement for informed consent was waived because the study used retrospective and de-identified clinical data.

### 4.2 Evaluation metrics

This study adopts Accuracy, Precision, Recall, and AUC as the main evaluation metrics to comprehensively assess the classification performance of the model in the sleep-related breathing event recognition task. Let *TP*, *TN*, *FP*, and *FN* denote true positives, true negatives, false positives, and false negatives, respectively, where positive samples indicate abnormal sleep-related breathing event segments and negative samples indicate normal sleep segments.


$$\begin{array}{l} \mathrm{Accuracy}=\frac{TP+TN}{TP+TN+FP+FN}. \end{array}$$ (21)

Accuracy is used to measure the overall classification correctness of the model on all test samples, reflecting its comprehensive recognition ability for both normal segments and abnormal respiratory event segments.


$$\begin{array}{l} \mathrm{Precision}=\frac{TP}{TP+FP}. \end{array}$$ (22)

Precision is used to measure the proportion of true abnormal events among the samples predicted by the model as abnormal respiratory events. A higher Precision indicates a lower probability of false alarms.


$$\begin{array}{l} \mathrm{Recall}=\frac{TP}{TP+FN}. \end{array}$$ (23)

Recall is used to measure the proportion of true abnormal respiratory events that are correctly detected by the model, reflecting the sensitivity of the model to abnormal sleep-related breathing segments.


$$\begin{array}{l} \mathrm{AUC}=\overset{1}{\underset{0}{\int }}\mathrm{TPR}\left(\mathrm{FPR}\right)d\mathrm{FPR}. \end{array}$$ (24)

AUC denotes the area under the ROC curve and is used to evaluate the ability of the model to distinguish normal segments from abnormal respiratory event segments under different classification thresholds. A higher AUC value indicates stronger overall discriminative performance.

## 5 Experimental results and analysis

### 5.1 Experimental setup

As shown in table 3 patient-level data partitioning and training protocol. Patient-level five-fold cross-validation is adopted for both the public and clinical datasets, with all segments from the same patient retained in a single fold. In each outer cross-validation round, one fold is held out exclusively for testing, while the patients in the remaining four folds are further divided into mutually exclusive training and validation subsets at the patient level. Thus, no patient contributes segments to both the training and validation subsets. The held-out test fold is not accessed during hyperparameter tuning, checkpoint selection, or model development. Identical outer-fold assignments are used for all comparison methods, and the final performance is obtained by aggregating the results from the five held-out test folds.

**Table 3:** Experimental environment and main training parameter settings.

Item	Setting
Operating system	Ubuntu 22.04 LTS
Deep learning framework	PyTorch
Computing device	4 NVIDIA H100 GPUs
Training epochs	100
Batch size	256
Optimizer	AdamW
Initial learning rate	1 × 10^−4^
Weight decay	5 × 10^−2^
Learning rate scheduler	Cosine Annealing
Embedding dimension	192
Encoder depth	8
Number of attention heads	6
Dropout	0.1
Mixed-precision training	Enabled
Arousal loss weight λ_arousal_	0.10
Stage loss weight λ_stage_	0.05
Focal focusing parameter γ	2.0
Class-balanced parameter β	0.999
Margin parameter δ	0.35
Ablation baseline	Dual-branch Vision-Mamba with direct feature concatenation
Cross-validation strategy	Patient-level five-fold cross-validation
Fold assignment	All segments from the same patient retained in the same fold
Checkpoint selection criterion	Validation Accuracy, predefined before test evaluation
Testing protocol	Each fold used as the held-out test set once
Comparison protocol	Identical fold assignments used for all methods

The recordings are divided into 30-s non-overlapping windows. Neurophysiological channels are identified by case-insensitive matching of EEG-, EOG-, EMG-, LOC-, ROC-, and CHIN-related EDF labels, with all matched channels retained in their original order. The neurophysiological signals are resampled to 128 Hz, independently standardized by z-score normalization within each window, and arranged as vertically separated waveform traces on a common 0–30 s time axis. Missing channels are skipped rather than imputed, and the resulting waveform images are resized to 224 × 224. Tracheal and microphone signals are selected for the respiration-acoustic modality, resampled to 8 kHz, standardized using z-score normalization, and converted into log-magnitude spectrograms using a 512-sample analysis window with 384-sample overlap. Both waveform and spectrogram images are subsequently normalized using the ImageNet mean and standard deviation before being provided to the model.

The model consists of a dual-branch state-space encoder, a cross-state token routing module, an event query prototype fusion module, and multi-task prediction heads. For the ablation study, the Baseline is defined as a dual-branch Vision-Mamba model in which the two modalities are encoded separately and directly concatenated before prediction, without CSTR or EQPF. All variants use the same preprocessing, patient-level fold assignments, and training settings. AdamW and cosine annealing are used for optimization, with mixed-precision training enabled. Hyperparameters, including λ_stage_, are selected exclusively from the training and validation data. Validation Accuracy was predefined before any evaluation on the held-out test folds and was used consistently as the checkpoint-selection criterion across all folds and both datasets. This criterion provides a fixed and uniform model-selection rule for the binary segment-level classification task, while class imbalance is addressed during model optimization through the class-balanced focal margin loss. The checkpoint-selection criterion is not modified according to held-out test performance.

### 5.2 Experimental results compared with other models

To comprehensively evaluate the effectiveness of the proposed framework, comparative experiments are conducted with representative methods on both the public and clinical datasets. All methods are trained and evaluated using the same patient-level five-fold cross-validation protocol, and identical fold assignments are maintained across different methods to ensure a fair comparison. The comparison methods include task-specific sleep-related respiratory event recognition methods, sleep-staging models adapted to the present task, and general-purpose architectures used as architecture-transfer baselines. Accuracy, Precision, Recall, and AUROC are adopted as the evaluation metrics. The comparison results on the PSG-Audio public dataset are presented in Table 4.

**Table 4:** Comparison results of task-specific methods, adapted sleep-staging models, and general-purpose architecture-transfer baselines on the PSG-Audio public dataset.

Method	Acc	Precision	Recall	AUROC
ResNet50 (34)	76.79 ± 2.15	77.73 ± 1.96	75.64 ± 0.94	81.07 ± 1.13
DeepSleepNet (35)	82.88 ± 1.70	73.46 ± 1.77	76.83 ± 0.88	78.63 ± 2.18
TinySleepNet (36)	80.19 ± 0.52	73.95 ± 0.88	81.03 ± 1.41	78.84 ± 1.19
XSleepNet (37)	77.78 ± 1.76	73.46 ± 1.57	76.73 ± 1.23	84.95 ± 1.94
Vision-Transformer (38)	82.00 ± 1.18	81.90 ± 0.87	81.24 ± 1.89	82.24 ± 1.57
Swin-Transformer (39)	83.91 ± 1.76	79.71 ± 1.72	78.17 ± 0.65	81.45 ± 0.56
ConvNeXtV2 (40)	83.34 ± 0.56	73.72 ± 1.55	79.10 ± 1.58	82.08 ± 0.97
VMamba (41)	76.50 ± 0.60	81.57 ± 0.92	76.75 ± 1.73	78.56 ± 1.09
EfficientViM (42)	84.97 ± 1.41	74.60 ± 0.73	77.48 ± 0.49	78.90 ± 0.73
SynthSleepNet (43)	82.48 ± 0.75	78.47 ± 1.40	79.18 ± 1.99	85.58 ± 2.04
Clinical-Transformer (44)	80.12 ± 2.08	71.46 ± 1.69	73.30 ± 0.88	82.14 ± 0.88
Mamba-SDB (45)	83.91 ± 1.33	82.54 ± 1.14	76.59 ± 0.61	83.18 ± 0.80
MT-TASPPNet (18)	80.65 ± 1.80	74.83 ± 1.19	80.89 ± 2.00	86.86 ± 1.12
SleepNet (22)	78.98 ± 1.49	80.05 ± 0.57	82.61 ± 1.51	90.32 ± 1.04
Xception-based method (14)	82.58 ± 0.86	78.45 ± 1.93	81.03 ± 1.03	83.50 ± 1.92
**Ours**	**88.19** **±** **1.21**	**84.51** **±** **1.72**	**86.28** **±** **1.33**	**92.71** **±** **1.48**

All results are reported as mean ± standard deviation across the five patient-level cross-validation folds.

As shown in Table 4, the proposed method achieves the highest mean values across all four evaluation metrics on the PSG-Audio public dataset. Among the competing methods other than the proposed method, EfficientViM provides the strongest Accuracy, Mamba-SDB achieves the highest Precision, and SleepNet obtains the strongest Recall and AUROC. These results show that the relative strengths of the comparison methods vary across different evaluation dimensions, whereas the proposed framework maintains consistently leading classification correctness, positive predictive performance, event sensitivity, and threshold-independent discriminative ability. Under the patient-level five-fold cross-validation protocol, this overall performance pattern supports the effectiveness of jointly modeling neurophysiological and respiration-acoustic representations through the dual-branch state-space encoder, cross-state token routing, and event query prototype fusion modules.

To further examine the performance of the proposed method in real-world clinical sleep monitoring scenarios, the same comparison methods are evaluated on the clinical dataset using the identical patient-level five-fold cross-validation protocol. All segments belonging to the same patient are retained within the same fold, and the same fold assignments are applied to all methods. The comparison results in terms of Accuracy, Precision, Recall, and AUROC are presented in Table 5.

**Table 5:** Comparison results of task-specific methods, adapted sleep-staging models, and general-purpose architecture-transfer baselines on the clinical dataset.

Method	Acc	Precision	Recall	AUROC
ResNet50	72.08 ± 0.80	68.85 ± 0.77	73.79 ± 1.07	79.56 ± 1.25
DeepSleepNet	69.73 ± 1.47	71.13 ± 1.12	79.45 ± 1.25	82.17 ± 0.67
TinySleepNet	70.75 ± 1.33	69.76 ± 1.63	76.85 ± 0.89	80.34 ± 1.17
XSleepNet	71.44 ± 0.75	72.98 ± 1.17	81.19 ± 1.40	85.61 ± 0.72
Vision-Transformer	68.58 ± 1.29	74.97 ± 0.81	77.71 ± 1.44	86.45 ± 1.10
Swin-Transformer	70.27 ± 0.81	72.20 ± 1.04	82.26 ± 1.27	84.30 ± 0.76
ConvNeXtV2	72.70 ± 1.25	70.35 ± 0.69	75.49 ± 0.75	83.79 ± 1.49
VMamba	73.09 ± 0.90	75.37 ± 0.68	78.35 ± 1.40	87.36 ± 1.76
EfficientViM	69.29 ± 0.73	72.91 ± 0.87	74.18 ± 1.22	81.53 ± 1.26
SynthSleepNet	71.95 ± 1.11	74.10 ± 1.00	82.74 ± 1.30	88.72 ± 1.20
Clinical-Transformer	72.33 ± 1.03	76.17 ± 1.03	80.36 ± 1.42	88.98 ± 1.25
Mamba-SDB	70.70 ± 0.89	73.51 ± 1.13	82.50 ± 0.89	86.85 ± 1.30
MT-TASPPNet	71.31 ± 1.32	74.62 ± 1.07	81.62 ± 0.75	87.43 ± 1.33
SleepNet	72.49 ± 1.22	75.21 ± 0.95	82.25 ± 1.22	88.56 ± 0.78
Xception-based method	73.00 ± 1.08	76.03 ± 1.21	82.89 ± 1.18	89.37 ± 0.92
**Ours**	**74.33** **±** **1.21**	**77.51** **±** **1.62**	**84.33** **±** **1.48**	**90.51** **±** **1.82**

All results are reported as mean ± standard deviation across the five patient-level cross-validation folds.

As shown in Table 5, the proposed method achieves the highest mean values for Accuracy, Precision, Recall, and AUROC on the clinical dataset. Among the comparison methods, VMamba obtains the strongest Accuracy, Clinical-Transformer achieves the highest Precision, and the Xception-based method provides the strongest Recall and AUROC. The different performance rankings among the comparison methods indicate that their advantages are concentrated in different evaluation dimensions. In comparison, the proposed framework maintains the leading mean performance across all four metrics under the same patient-level cross-validation protocol, reflecting a more balanced ability to distinguish normal and abnormal respiratory event segments. This result suggests that the interaction between neurophysiological states and respiration-acoustic dynamics, together with event-aware prototype aggregation, remains effective when evaluated on clinical data with different signal characteristics and patient distributions.

### 5.3 Ablation test results

To further verify the effectiveness of each key component in the proposed model framework, this study conducts systematic ablation experiments by removing the cross-state token routing module, the event query prototype fusion module, or the class-balanced focal margin loss from the complete model. All model variants use the same data partitions, training parameters, and evaluation metrics to ensure a fair comparison. The experimental results are presented in Table 6.

**Table 6:** All results are reported as mean ± standard deviation across the five patient-level cross-validation folds.

Dataset	Method	Acc	Precision	Recall	AUROC
Public dataset	Baseline	81.68 ± 0.72	76.93 ± 0.76	78.62 ± 0.64	84.61 ± 1.18
	W/o CSTR	84.09 ± 1.04	82.81 ± 0.71	83.47 ± 1.42	89.66 ± 1.37
	W/o EQPF	86.52 ± 1.17	81.96 ± 1.43	81.81 ± 0.98	91.11 ± 1.32
	W/o CBFML	85.23 ± 0.91	80.65 ± 0.94	84.67 ± 1.33	88.22 ± 0.92
	**Ours**	**88.19** **±** **1.21**	**84.51** **±** **1.72**	**86.28** **±** **1.33**	**92.71** **±** **1.48**
Clinical dataset	Baseline	67.37 ± 1.22	69.37 ± 0.78	75.73 ± 1.33	80.29 ± 1.05
	W/o CSTR	72.69 ± 1.36	73.46 ± 1.14	82.52 ± 1.51	85.46 ± 1.48
	W/o EQPF	70.27 ± 1.37	75.55 ± 1.64	81.43 ± 1.48	87.69 ± 1.34
	W/o CBFML	71.61 ± 1.04	74.79 ± 1.18	79.83 ± 0.80	89.16 ± 0.81
	**Ours**	**74.33** **±** **1.21**	**77.51** **±** **1.62**	**84.33** **±** **1.48**	**90.51** **±** **1.82**

As shown in Table 6, the complete model achieves the best performance across all four metrics on both datasets. Compared with the Baseline, it improves Acc, Precision, Recall, and AUROC by 6.51, 7.58, 7.66, and 8.10 percentage points, respectively, on the public dataset, with corresponding improvements of 6.96, 8.14, 8.60, and 10.22 percentage points on the clinical dataset. Removing CSTR causes particularly evident reductions in Accuracy on the public dataset and in Precision and AUROC on the clinical dataset, demonstrating the importance of bidirectional cross-modal interaction. Removing EQPF produces the largest Recall reduction on the public dataset and the largest Accuracy reduction on the clinical dataset, indicating its contribution to event-related feature aggregation and overall classification correctness. Removing CBFML leads to the largest public-dataset AUROC reduction and the largest clinical-dataset Recall reduction, supporting its effectiveness in improving discriminative robustness and abnormal-event sensitivity. These results demonstrate that CSTR, EQPF, and CBFML provide complementary contributions to the complete framework.

### 5.4 Confusion matrix experimental results

**Figure 4:**
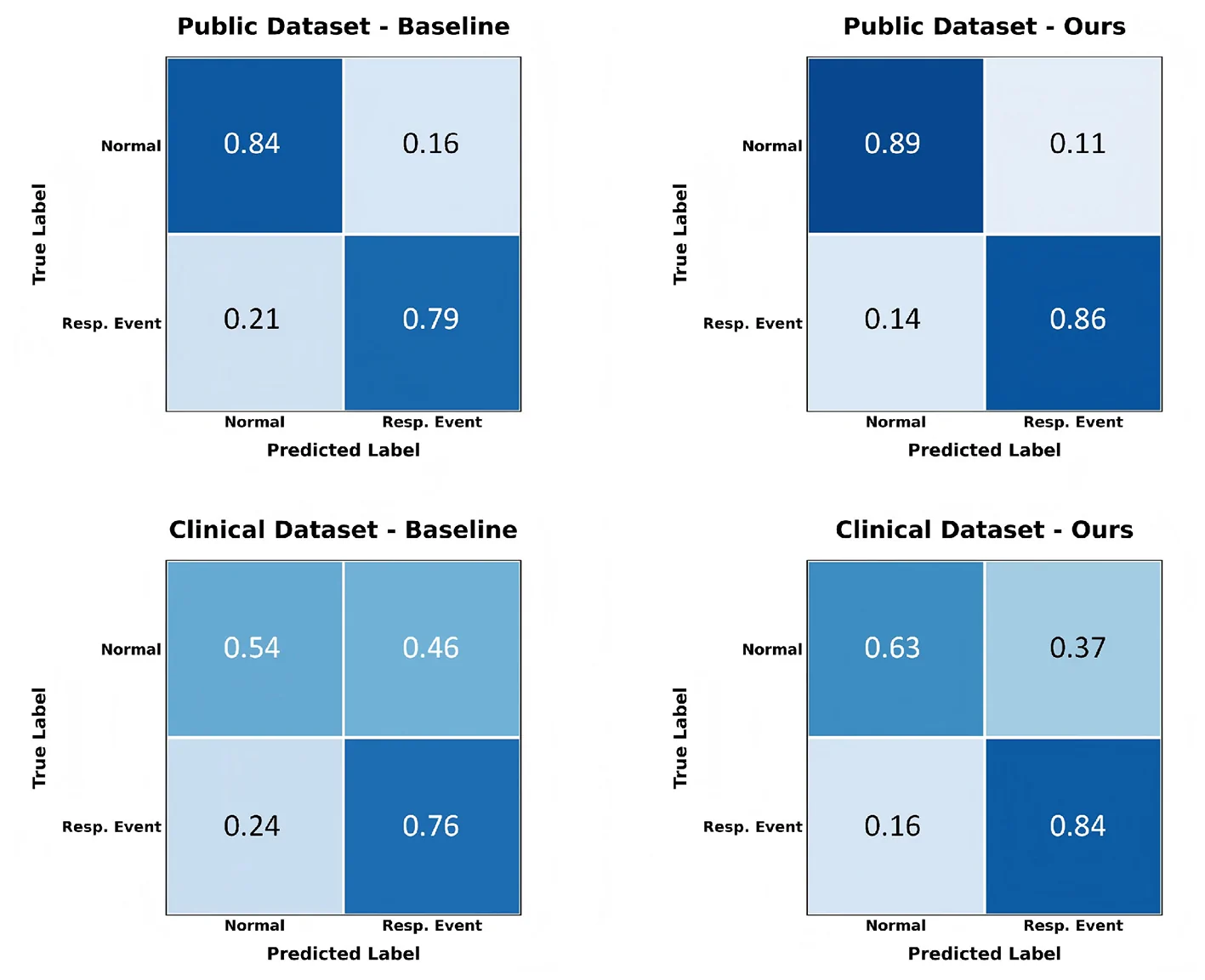
Normalized confusion matrices of the Baseline and the proposed method on the public and clinical datasets, illustrated using one representative test fold from the patient-level five-fold cross-validation.

To further examine the class-wise discriminative behavior of the model, this study presents confusion matrices to visualize the prediction distribution and misclassification patterns for normal segments and respiratory event segments. Since all experiments are conducted under the patient-level five-fold cross-validation protocol, the confusion matrices shown in Figure 4 are derived from one representative test fold and are used as a qualitative illustration of the classification characteristics of the Baseline and the proposed method on both the public and clinical datasets. Through this visualization, the class discrimination tendency of the model can be observed more intuitively, thereby providing supplementary evidence for the interpretation of the comparative experimental results.

As shown in Figure 4, the proposed method exhibits a more concentrated diagonal distribution than the Baseline on both datasets, suggesting a clearer separation between normal segments and respiratory event segments. Relative to the Baseline, the proposed method shows fewer off-diagonal confusions in both class directions, indicating a more balanced recognition behavior for the two categories. This tendency is more evident on the public dataset, where the class boundaries appear relatively clearer. On the clinical dataset, the confusion patterns remain more pronounced, which suggests that real-world clinical data involve greater variability and a higher degree of recognition difficulty. Even so, the proposed method still maintains a more coherent confusion structure than the Baseline, which is consistent with the overall improvement observed in the quantitative comparison results.

### 5.5 t-SNE experimental results

**Figure 5:**
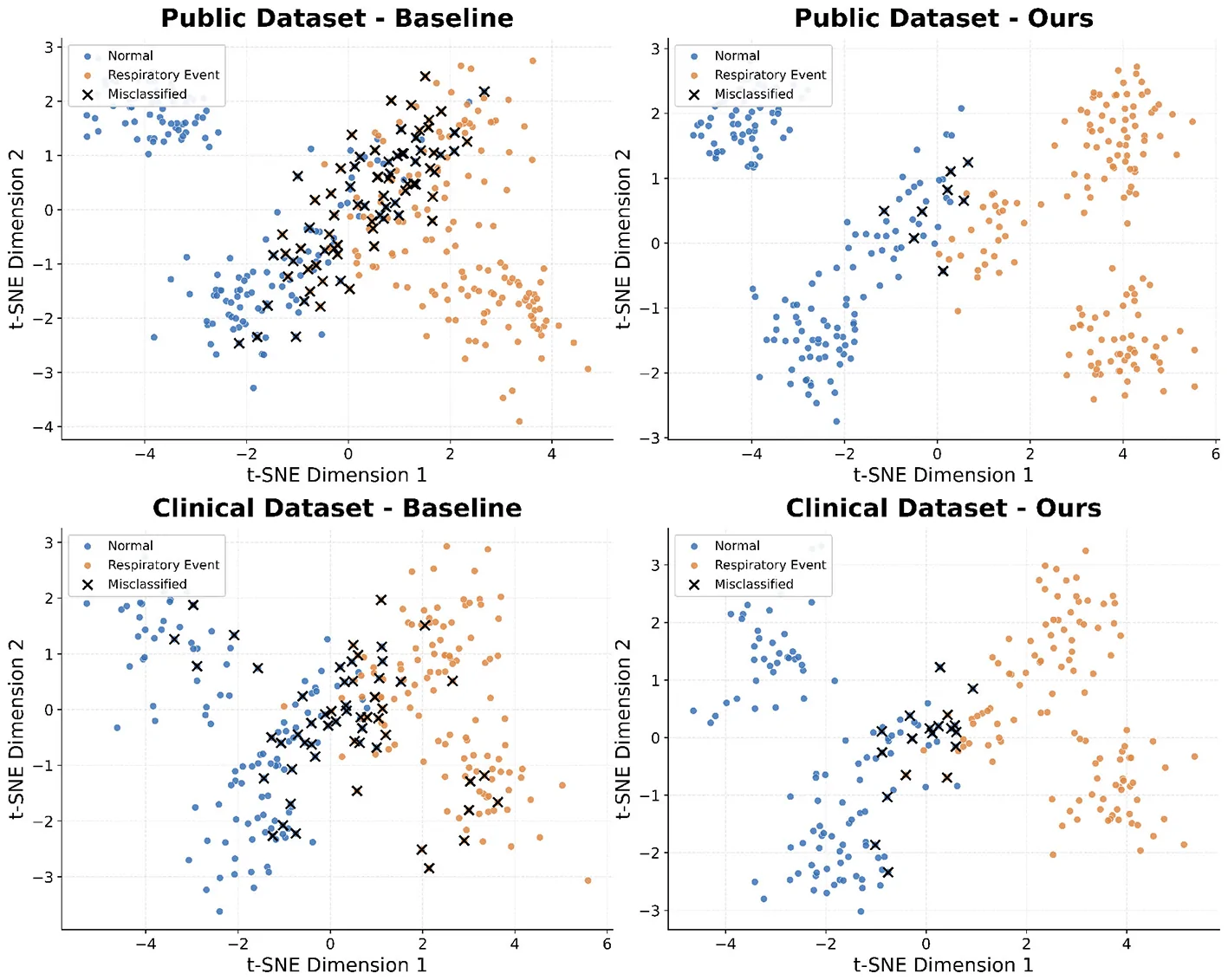
t-SNE visualization of the feature distributions learned by the Baseline and the proposed method on the public and clinical datasets, illustrated using one representative test fold from the patient-level five-fold cross-validation.

To further examine the organization of the high-dimensional representations learned by the models, this study employs t-SNE to project the deep features of test samples into a two-dimensional space. Under the patient-level five-fold cross-validation protocol, the visualization presented in Figure 5 is generated using the test samples from one representative fold, with the same fold used for both the Baseline and the proposed method to ensure a consistent qualitative comparison. Normal segments and respiratory event segments are represented by different colors, while misclassified samples are additionally marked to illustrate their locations in the projected feature space. This visualization is intended to provide complementary qualitative evidence regarding intra-class clustering, inter-class separation, and the distribution of difficult samples.

As shown in Figure 5, the feature representations learned by the Baseline exhibit noticeable overlap between normal segments and respiratory event segments, particularly in the transition regions between the two classes. In comparison, the proposed method produces more compact within-class distributions and clearer separation between the two categories on both datasets, while most misclassified samples remain concentrated near the class-boundary regions. The separation tendency is more distinct on the public dataset, whereas the clinical dataset presents a more complex feature distribution. Nevertheless, the proposed method still demonstrates a more organized representation structure than the Baseline on the clinical data. These visualization patterns are consistent with the quantitative comparison results and support the interpretation that cross-modal state interaction and event-aware feature aggregation contribute to improved class separability. Since the visualization is based on one representative cross-validation fold and a nonlinear two-dimensional projection, it is used as qualitative supplementary evidence rather than an independent quantitative evaluation.

### 5.6 AUROC experimental results

**Figure 6:**
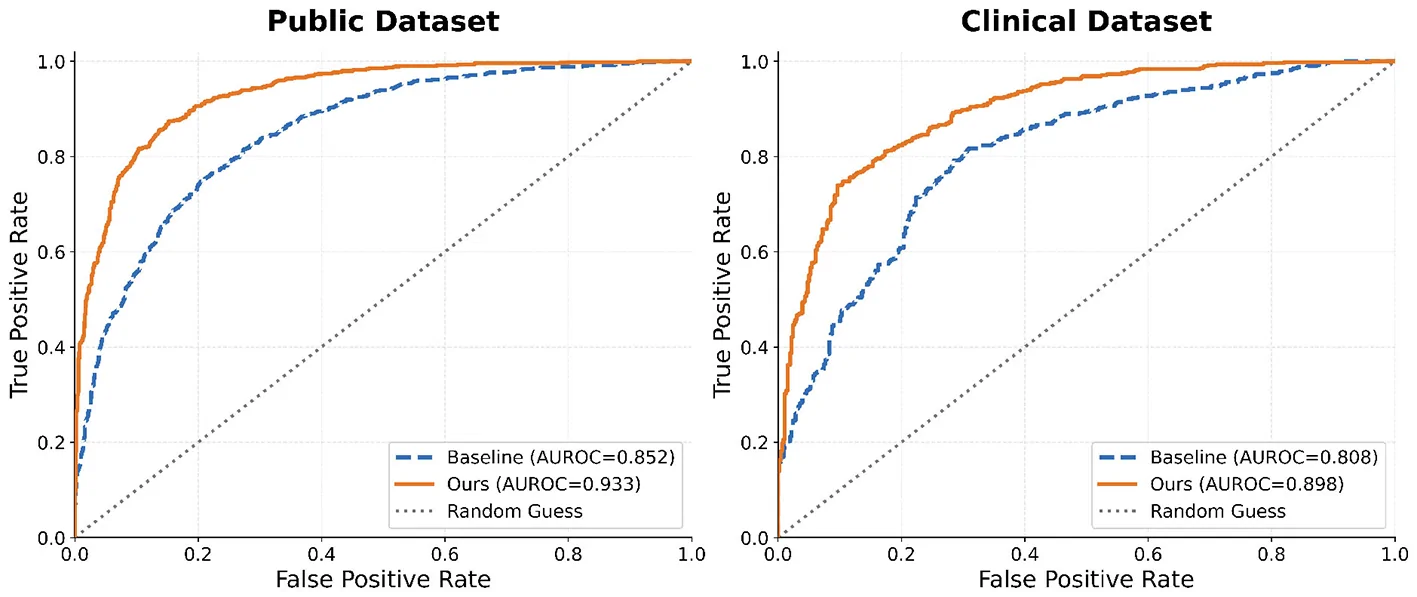
ROC curves of the Baseline and the proposed method on the public and clinical datasets, illustrated using one representative test fold from the patient-level five-fold cross-validation. The AUROC values shown in the legends are calculated from the corresponding representative fold.

To further evaluate the threshold-dependent discriminative behavior of the model, this study plots the ROC curves of the Baseline and the proposed method on both the public and clinical datasets. Under the patient-level five-fold cross-validation protocol, the curves presented in Figure 6 are generated from the test predictions of one representative fold, with the same fold used for both methods to ensure a consistent comparison. The AUROC values displayed in the figure legend therefore correspond specifically to this representative test fold. The visualization is used to examine the relationship between the true positive rate and false positive rate under different decision thresholds and to provide qualitative supplementary evidence for the probabilistic ranking ability of the models.

As shown in Figure 6, the ROC curve of the proposed method is generally positioned above that of the Baseline and closer to the upper-left region on both datasets. This pattern indicates that the proposed method maintains a higher true positive rate across most comparable false positive rate ranges and exhibits stronger probabilistic ranking ability on the selected test fold. The separation between the two curves is more apparent on the public dataset, while the clinical dataset presents a comparatively more challenging discrimination pattern. Nevertheless, the proposed method retains a consistent advantage over the Baseline across the principal threshold range on the clinical data. These fold-level observations are consistent with the aggregate five-fold cross-validation results and provide complementary visual evidence for the overall discriminative performance of the proposed framework.

### 5.7 Statistical significance analysis against a task-specific external competitor

**Figure 7:**
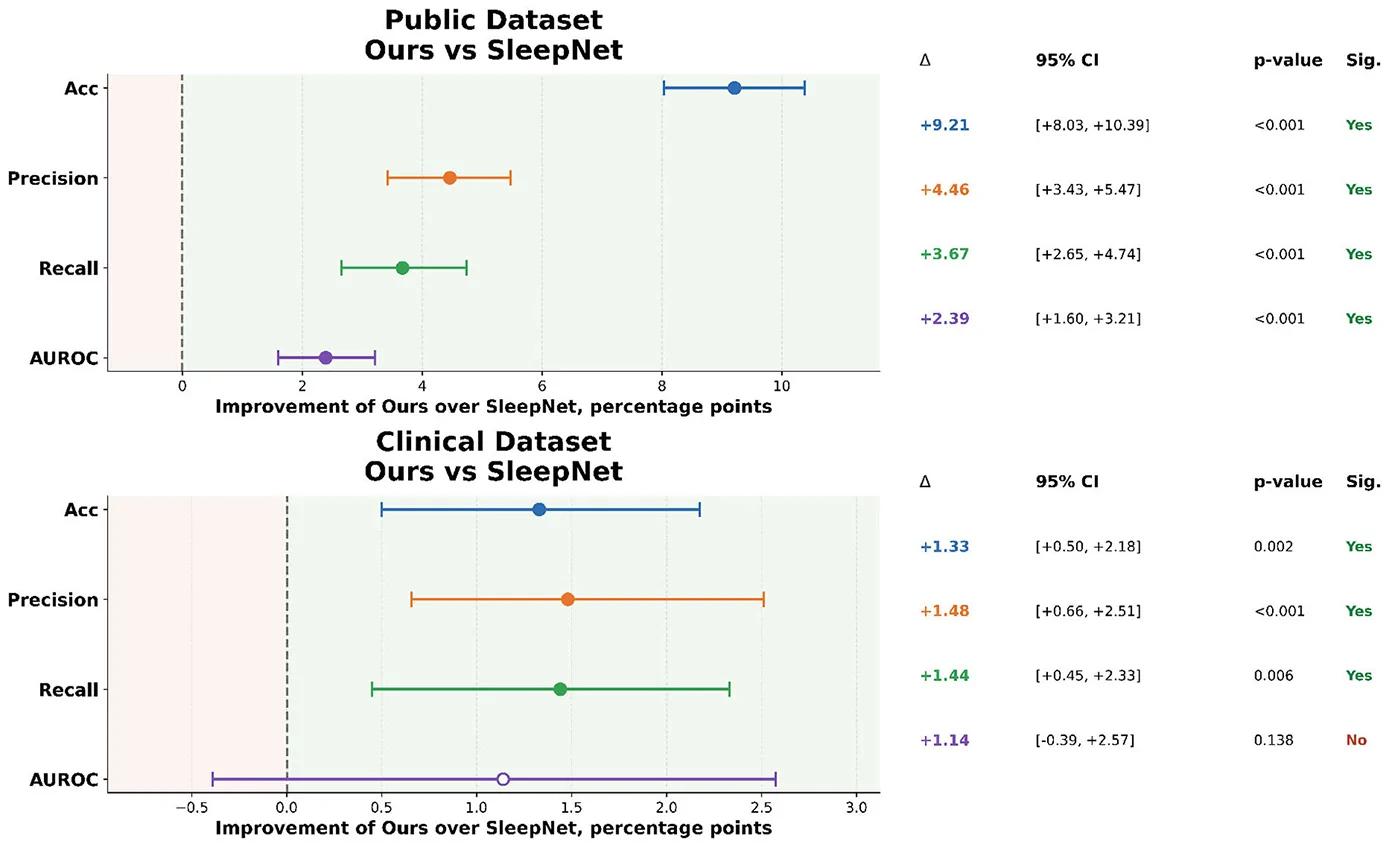
Patient-level paired bootstrap analysis of the performance differences between the proposed method and SleepNet on the public and clinical datasets. The analysis is based on the pooled out-of-fold predictions obtained from all five test folds of the patient-level five-fold cross-validation. Each patient contributes predictions only from the fold in which that patient was held out for testing. The dots denote the mean paired performance differences in percentage points, the horizontal lines represent the corresponding 95% confidence intervals, and the dashed vertical line indicates zero difference.

To further examine whether the performance differences between the proposed method and a strong task-specific external competitor are statistically supported, this study performs a patient-level paired bootstrap analysis based on the pooled out-of-fold predictions obtained from the complete patient-level five-fold cross-validation. SleepNet is selected as the reference method for both the public and clinical datasets because it demonstrates strong overall performance among the task-specific comparison methods, particularly in terms of event sensitivity and threshold-independent discrimination. In the five-fold cross-validation procedure, each patient is included in the test set exactly once, and the corresponding predictions are produced by a model that has not been trained using data from that patient. The out-of-fold predictions from all five test folds are subsequently pooled to construct the complete evaluation set. Since multiple 30-s segments may originate from the same patient, bootstrap resampling is conducted at the patient level rather than at the segment level. In each bootstrap iteration, patients are sampled with replacement from the pooled out-of-fold evaluation set, and all segments belonging to each sampled patient are retained. The same resampled patients are used for the proposed method and SleepNet to preserve the paired comparison. Accuracy, Precision, Recall, and AUROC are recalculated for both methods in each bootstrap sample, and their paired differences are used to estimate the mean differences, 95% confidence intervals, and corresponding *p*-values. Because this bootstrap analysis is calculated from the pooled out-of-fold predictions, whereas the comparison tables report the unweighted mean and standard deviation of the five fold-level metrics, the mean differences shown in this analysis are not necessarily identical to the direct differences between the fold-averaged values reported in the tables. The results are presented in Figure 7.

As shown in Figure 7, the proposed method exhibits positive mean differences relative to SleepNet across all evaluated metrics on both datasets. On the public dataset, the confidence intervals for all four metrics remain above the zero-difference reference, providing statistical support for the observed improvements. On the clinical dataset, the confidence intervals for Accuracy, Precision, and Recall also remain above zero, providing statistical support for the corresponding improvements, whereas the confidence interval for the AUROC difference crosses zero and therefore does not provide sufficient evidence of a statistically significant AUROC improvement. Overall, the patient-level paired bootstrap analysis based on the pooled out-of-fold predictions from all five test folds provides supplementary statistical evidence for the improvements in classification correctness and respiratory-event recognition. However, the evidence for improved threshold-independent discrimination on the clinical dataset remains inconclusive, and the results should not be interpreted as uniform evidence of superiority across all metrics.

### 5.8 Predictive uncertainty distribution analysis

**Figure 8:**
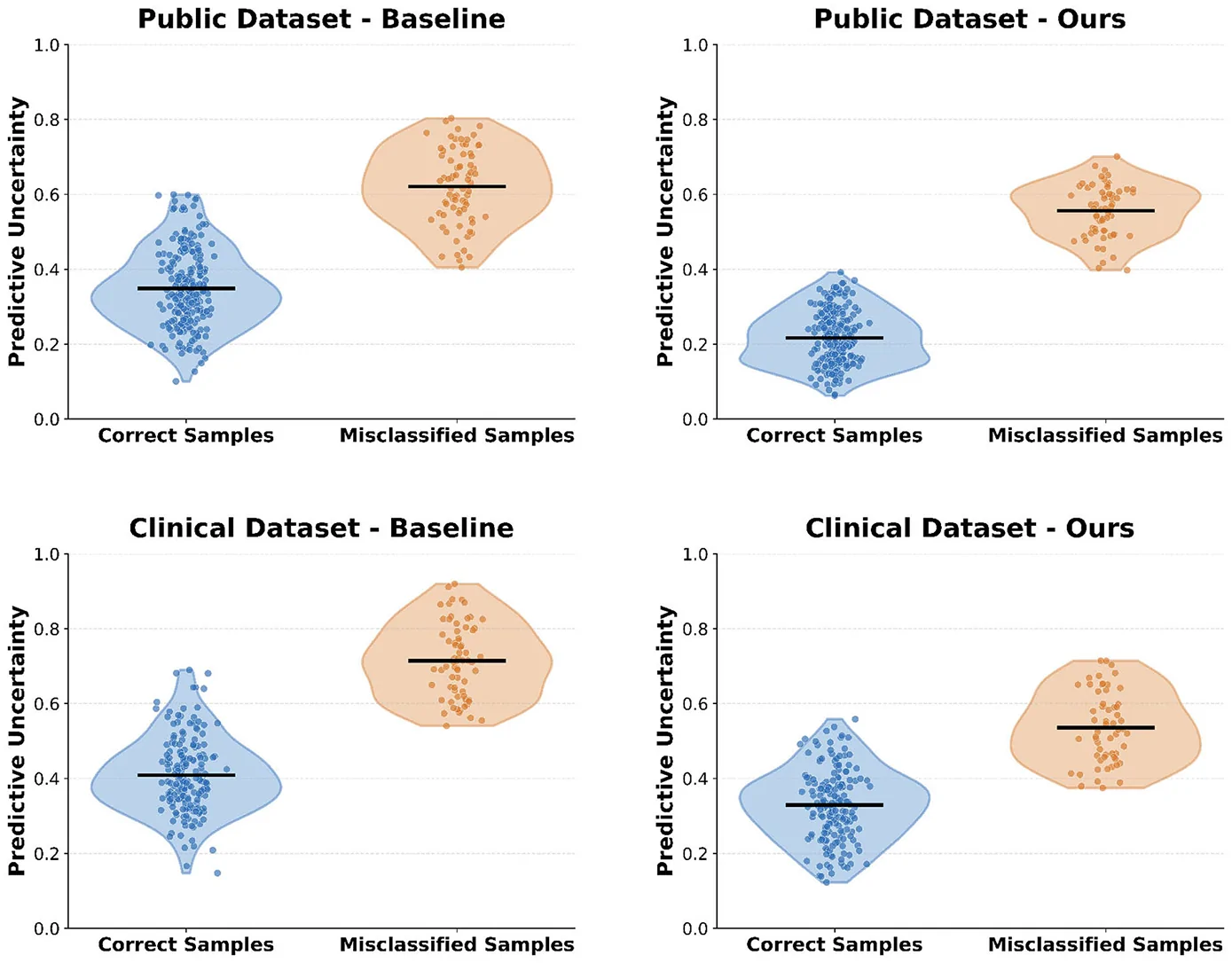
Predictive uncertainty distributions of correctly classified and misclassified samples for the Baseline and the proposed method on the public and clinical datasets, illustrated using one representative test fold from the patient-level five-fold cross-validation.

To further examine the confidence behavior of the model under different prediction outcomes, this study visualizes the predictive uncertainty distributions of correctly classified and misclassified samples for both the Baseline and the proposed method. Under the patient-level five-fold cross-validation protocol, the results presented in Figure 8 are obtained from the test predictions of one representative fold, with the same fold used for both methods to ensure a consistent qualitative comparison. The violin plots describe the distribution of the uncertainty scores, the individual points represent test samples, and the horizontal lines indicate the central tendency of each distribution. This analysis is used as supplementary evidence to observe whether prediction errors are associated with greater model uncertainty.

As shown in Figure 8, correctly classified samples generally exhibit lower predictive uncertainty than misclassified samples for both methods and on both datasets, indicating that erroneous predictions tend to be accompanied by greater model hesitation. Compared with the Baseline, the proposed method presents lower and more concentrated uncertainty distributions for correctly classified samples, while the uncertainty distribution of misclassified samples is also shifted downward. The clinical dataset exhibits higher overall uncertainty than the public dataset, consistent with its more complex prediction patterns observed in the preceding analyses. Nevertheless, the distinction between correctly classified and misclassified samples remains visible for the proposed method on the clinical data. These observations suggest that the proposed framework produces a more organized confidence pattern within the selected test fold, although the figure should be interpreted as qualitative fold-level evidence rather than as an aggregate uncertainty assessment across all five folds.

### 5.9 Auxiliary sleep-stage loss weight sensitivity analysis

To investigate the influence of sleep-stage auxiliary supervision on the proposed framework, this study evaluates different values of λ_stage_ on both the public and clinical datasets under the patient-level five-fold cross-validation protocol. The value λ_stage_ = 0.05 used in the main experiments was selected exclusively using the training and validation data within the development folds and was fixed before evaluation on the held-out outer test folds. The outer test folds were not used for hyperparameter selection or model configuration. Except for the sleep-stage auxiliary loss weight, all model configurations, training parameters, and fold assignments are kept unchanged to ensure a controlled comparison. The purpose of this experiment is to examine whether the proposed framework maintains stable performance under different levels of auxiliary supervision and to determine an appropriate balance between respiratory event discrimination and sleep-stage context modeling. The results for the remaining candidate values are reported only as a supplementary robustness analysis and were not used to retrospectively modify the hyperparameter setting adopted in the main experiments. The experimental results are presented in Table 7.

**Table 7:** Sensitivity analysis of the sleep-stage auxiliary loss weight λ_stage_ on the public and clinical datasets.

Dataset	λ_stage_	Acc	Precision	Recall	AUROC
Public dataset	0.03	84.62 ± 1.40	80.43 ± 0.70	82.91 ± 1.00	88.33 ± 1.60
	**0.05**	**88.19** **±** **1.21**	**84.51** **±** **1.72**	**86.28** **±** **1.33**	**92.71** **±** **1.48**
	0.10	86.44 ± 1.44	82.68 ± 1.51	84.16 ± 1.20	91.38 ± 1.05
	0.20	83.44 ± 1.28	81.76 ± 1.02	84.61 ± 1.37	89.16 ± 1.55
	0.35	85.53 ± 1.45	78.94 ± 1.21	80.75 ± 1.06	87.38 ± 1.43
	0.50	81.76 ± 1.22	82.07 ± 1.67	78.58 ± 0.66	85.72 ± 0.71
Clinical dataset	0.03	71.43 ± 0.82	74.20 ± 1.21	80.26 ± 1.26	86.32 ± 1.85
	**0.05**	**74.33** **±** **1.21**	**77.51** **±** **1.62**	**84.33** **±** **1.48**	**90.51** **±** **1.82**
	0.10	72.82 ± 0.53	75.50 ± 1.62	82.58 ± 1.44	88.19 ± 0.74
	0.20	69.39 ± 0.79	71.90 ± 1.33	81.34 ± 1.49	88.52 ± 1.64
	0.35	73.21 ± 0.62	75.79 ± 1.41	82.36 ± 1.63	85.51 ± 1.30
	0.50	68.54 ± 0.81	72.72 ± 1.59	77.72 ± 1.57	87.33 ± 1.50

All results are reported as mean ± standard deviation across the five patient-level cross-validation folds. Bold values indicates the optimal result.

As shown in Table 7, the proposed framework maintains generally stable training and evaluation behavior across the examined auxiliary loss weights, without exhibiting abrupt performance collapse under moderate changes in λ_stage_. The relatively controlled fold-wise variations further indicate that the model retains a certain degree of robustness to the selection of this hyperparameter. Nevertheless, the mean performance does not improve monotonically as the auxiliary supervision weight increases. A relatively small weight may provide insufficient sleep-stage contextual guidance, whereas an excessively large weight may cause the auxiliary task to exert excessive influence on the shared representation and weaken the optimization focus of the primary respiratory event recognition task. A moderate setting produces the most balanced performance across classification correctness, precision, event sensitivity, and threshold-independent discrimination on both datasets.

## 6 Discussion

**Figure A1:**
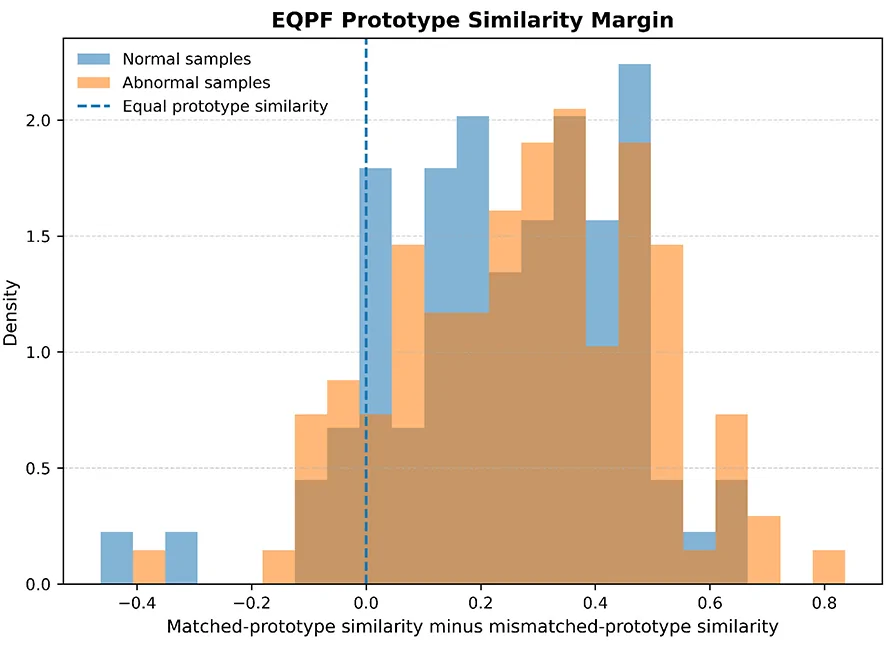
Distribution of the EQPF prototype similarity margins for normal and abnormal samples, where a positive margin indicates greater similarity to the matched class prototype than to the mismatched prototype.

This study develops a multimodal physiological representation learning framework for sleep-related breathing event recognition by jointly modeling neurophysiological state information and respiration-acoustic dynamics. Through cross-state token routing, the proposed framework enables fine-grained bidirectional interaction between the two modalities, while event query prototype fusion further aggregates respiratory-event-related information from the multimodal temporal representations. The gated cross-attention and learnable-query operations used in CSTR and EQPF are grounded in established multimodal and query-based representation learning mechanisms (10, 11), whereas the contribution of this study lies in adapting and combining these mechanisms for symmetric physiological-state routing and respiratory-event-oriented temporal aggregation. Additional analyses of performance under different AHI thresholds and the EQPF prototype-similarity margin distributions are provided in Table A1 and Figure A1, respectively. The patient-level paired bootstrap analysis further supports several of the observed performance improvements; however, statistical superiority is not uniform across all metrics and datasets, particularly because the AUROC difference on the clinical dataset does not reach statistical significance. Within the evaluated cohorts, the proposed method shows potential as a supplementary tool for automated polysomnographic analysis and auxiliary annotation, but the current evidence is insufficient to establish its effectiveness as an independent clinical screening or diagnostic system.

The proposed model contains 9.59 M trainable parameters. To further characterize its computational requirements, inference efficiency was evaluated using three different single-GPU configurations while keeping the model architecture and parameter count unchanged. As summarized in Table 8, the average inference time per 30-s segment was 16.37 ms on an NVIDIA H100 GPU, 28.71 ms on an NVIDIA GeForce RTX 4090 GPU, and 49.77 ms on an NVIDIA GeForce RTX 2080 GPU. These results indicate that inference can be performed on a single GPU without requiring the four-H100 configuration used for model training. CPU-based inference was not evaluated in this study.

**Table 8:** Inference efficiency of the proposed model under different single-GPU configurations.

Inference hardware	Parameters (M)	Inference time (ms/segment)
NVIDIA H100	9.59	16.37
NVIDIA GeForce RTX 4090	9.59	28.71
NVIDIA GeForce RTX 2080	9.59	49.77

The model architecture and number of trainable parameters remain unchanged across all hardware settings.

Several limitations should nevertheless be considered. First, the use of 30-s non-overlapping segments is consistent with standard sleep-scoring epochs and provides fixed-length multimodal inputs, but respiratory events may span adjacent segment boundaries and consequently introduce label ambiguity or incomplete temporal representation. Future work may therefore investigate overlapping windows or event-level temporal modeling. Second, the clinical dataset contains only 83 patients from a single medical center. Although patient-level five-fold cross-validation reduces dependence on a single train–test split, it cannot eliminate the limitations associated with the modest cohort size and potential single-center bias. Variations in acquisition equipment, electrode placement, patient characteristics, signal quality, and clinical workflow may influence model transferability across institutions. Accordingly, the present clinical results should be interpreted as evidence of feasibility and potential within the evaluated cohort rather than as validation of routine clinical applicability, deployment readiness, or broad clinical generalizability. The additional AHI-threshold analyses provide complementary retrospective evidence regarding model behavior across clinically meaningful severity thresholds, but they are not equivalent to prospective clinical validation.

Furthermore, the proposed model was not directly compared with the independent judgments of individual clinical experts on the same out-of-fold evaluation samples. Therefore, the present study cannot determine whether its performance reaches, exceeds, or remains below expert-level performance, and no claim of equivalence to or replacement of professional clinical judgment is made. Clinical signal missingness, artifacts, and uncertainty in respiratory-event boundaries may also affect prediction reliability. Future studies should therefore include larger independent multi-center cohorts, prospective validation, direct model–expert comparison, signal-quality assessment, cross-center domain adaptation, and more fine-grained event-level modeling to further evaluate the reliability, generalizability, and practical value of the proposed framework.

## 7 Conclusion

This study proposes a multimodal state-space representation learning framework for sleep-related breathing event recognition. By jointly modeling the neurophysiological signal feature matrix and the respiration-acoustic-related feature matrix, the proposed framework achieves effective discrimination of sleep-related breathing events. Methodologically, this study constructs a dual-branch state-space encoding structure to extract sequential dynamic features from different modalities, and further designs a cross-state token routing module and an event query prototype fusion module to enhance inter-modal information interaction and event-related representation aggregation. Meanwhile, the class-balanced focal margin loss, together with auxiliary supervision from sleep stages and arousal states, is introduced into the training objective to improve the model's adaptability to complex samples and imbalanced event distributions. Experimental results obtained under patient-level five-fold cross-validation show that the proposed method achieves competitive overall performance on both the public and clinical datasets, while the comparative experiments, ablation studies, and visualization analyses provide supporting evidence for the contributions of the proposed components. The patient-level paired bootstrap analysis based on the pooled out-of-fold predictions obtained from all five held-out test folds provides supplementary statistical evidence for several performance differences, while the AUROC improvement on the clinical dataset does not reach statistical significance. These statistical findings are therefore interpreted as supplementary evidence derived from the complete out-of-fold evaluation set rather than as uniform proof of superiority across all evaluation metrics and conditions. Overall, the proposed method provides a multimodal modeling strategy for sleep physiological signal analysis and shows preliminary potential as an auxiliary reference for sleep-related breathing event recognition within the evaluated cohorts. Given the limited single-center clinical sample and the absence of a direct comparison with individual clinical experts, further independent, multi-center, and prospective validation is necessary before its clinical utility or broader generalizability can be established.

## Data Availability

The original contributions presented in the study are included in the article/supplementary material, further inquiries can be directed to the corresponding authors.

## Appendix

**Table A1:** Different AHI experimental results.

Dataset	AHI Threshold	Sensitivity (%)	Specificity (%)	Accuracy (%)	AUROC (%)
Clinical dataset	AHI ≥ 5	81.94 ± 3.12	70.00 ± 4.56	79.56 ± 2.85	81.24 ± 3.59
	AHI ≥ 15	82.56 ± 6.13	75.71 ± 5.87	79.49 ± 3.49	84.13 ± 3.38
	AHI ≥ 30	79.67 ± 2.98	84.39 ± 4.14	83.09 ± 2.93	86.90 ± 2.55
Public dataset	AHI ≥ 5	95.50 ± 1.76	75.24 ± 3.85	95.20 ± 1.81	91.00 ± 1.16
	AHI ≥ 15	92.69 ± 0.79	85.44 ± 4.37	92.34 ± 0.83	92.38 ± 0.91
	AHI ≥ 30	89.49 ± 3.33	89.52 ± 1.96	89.49 ± 2.92	94.47 ± 0.71
